# Development of Objective Measurements of Scratching as a Proxy of Atopic Dermatitis—A Review

**DOI:** 10.3390/s25144316

**Published:** 2025-07-10

**Authors:** Cheuk-Yan Au, Neha Manazir, Huzhaorui Kang, Ali Asgar Saleem Bhagat

**Affiliations:** 1 Institute for Health Innovation & Technology (iHealthtech), National University of Singapore (NUS), MD6, 14 Medical Drive, #14-01, Singapore 117599, Singapore; cy.au@nus.edu.sg (C.-Y.A.); huzhaorui.kang@u.nus.edu (H.K.); 2 Department of Biomedical Engineering, National University of Singapore (NUS), 4 Engineering Drive 3, Singapore 117583, Singapore

**Keywords:** atopic dermatitis, dermatology, scratching, wearable sensors, medical device, neural network

## Abstract

Eczema, or atopic dermatitis (AD), is a chronic inflammatory skin condition characterized by persistent itching and scratching, significantly impacting patients’ quality of life. Effective monitoring of scratching behaviour is crucial for assessing disease severity, treatment efficacy, and understanding the relationship between itch and sleep disturbances. This review explores current technological approaches for detecting and monitoring scratching and itching in AD patients, categorising them into contact-based and non-contact-based methods. Contact-based methods primarily involve wearable sensors, such as accelerometers, electromyography (EMG), and piezoelectric sensors, which track limb movements and muscle activity associated with scratching. Non-contact methods include video-based motion tracking, thermal imaging, and acoustic analysis, commonly employed in sleep clinics and controlled environments to assess nocturnal scratching. Furthermore, emerging artificial intelligence (AI)-driven approaches leveraging machine learning for automated scratch detection are discussed. The advantages, limitations, and validation challenges of these technologies, including accuracy, user comfort, data privacy, and real-world applicability, are critically analysed. Finally, we outline future research directions, emphasizing the integration of multimodal monitoring, real-time data analysis, and patient-centric wearable solutions to improve disease management. This review serves as a comprehensive resource for clinicians, researchers, and technology developers seeking to advance objective itch and scratch monitoring in AD patients.

## 1. Introduction

Atopic dermatitis (AD), commonly referred to as eczema, is a chronic inflammatory skin disorder that affects approximately 1 in 10 individuals during their lifetime. Being the most common skin disease, it affects around 1 in 5 children and around 1 in 10 adults worldwide [1]. A hallmark symptom of AD is pruritus, an itchy and unpleasant sensation that makes patients have the urge to scratch. However, alleviation of pruritus via scratching could compromise the skin barrier, facilitating infection and exacerbating inflammation, perpetuating the ’itch–scratch cycle’ that negatively impacts patients’ quality of life [2]. Persistent itch and sleep disturbance caused by itching are significant contributors to disease burden [3]. Additionally, the visibility of skin lesions can lead to social stigma and emotional distress; the chronic nature of the condition is also associated with increased rates of anxiety and depression.

Traditionally, AD severity has been evaluated using clinical tools such as the Severity Scoring of Atopic Dermatitis (SCORAD) [4], Eczema Area and Severity Index (EASI) [5], Peak Pruritus Numerical Rating Scale (PP-NRS) [6], and Visual Analogue Scale (VAS) [7]. These methods integrate patient self-reporting of symptoms, including itch intensity over specific time frames, with clinical evaluation of signs like erythema, inflammation, induration, and affected body surface area using standardized scoring systems to evaluate disease severity and extent. While valuable to gain insights into the patients’ conditions, these qualitative instruments lack objectivity, making them liable to systemic errors or subjective opinions. They also offer limited insight into their frequency, duration, or itch intensity, factors that would be useful for monitoring disease progression and assessing therapeutic outcomes [8]. This is especially problematic in populations such as children or during nighttime episodes when self-reporting is inconsistent or impossible. This could result in suboptimal treatment being provided to patients as clinicians rely on the aforementioned clinical tools for prescribing treatment and dispensing medication [9].

Scratching behaviour has long been recognized as a valid and objective proxy for itch and disease severity in atopic dermatitis [10]. It is an observable and quantifiable behaviour that is temporally linked to episodes of pruritus, offering real-time insight into disease activity. Video recording with manual annotation of scratching by trained observers has been the recognised gold standard for objective assessment in AD [11]. Although proven effective for monitoring patterns and location of scratching, it is also very labour-intensive and there are concerns about privacy; therefore, it is impractical for routine use in clinical settings [10]. At the turn of the century, research has increasingly focused on objective, technology-driven approaches for measuring scratching as a proxy for itch. This includes a range of sensor-based techniques—such as accelerometers, acoustic sensors, strain gauges, pressure sensors, and vibratory detectors—that are not constrained to specific body locations [12]. More recently, there has been a growing emphasis on location-agnostic wearables or non-contact systems. These devices offer scalable and efficient solutions for continuous scratch monitoring [13], offering promising pathways to improve disease monitoring, personalise treatment, and optimize clinical decision-making in AD.

This review aims to survey and critically evaluate the current developmental landscape of scratch measuring technologies, highlighting their methodology and directions for integration into clinical care.

## 2. Search Strategy

The key theme of this literature review is “devices that measure scratching as an objective proxy to the severity of atopic dermatitis, eczema, or other skin conditions”. Review articles, articles that focused on devices intended for, or consisting of studies with non-human subjects, as well as other intervention studies using scratch measurement devices either as a comparison or feedback were excluded.

We have queried PubMed, Scopus, and Google Scholar for articles dated from January 2015 to March 2025. The queried terms are listed below:

**SCOPUS**  (TITLE-ABS-KEY(“scratching” OR “objective measurement” OR “quantify”) AND TITLE-ABS-KEY(“pruritus” OR “Atopic Dermatitis” OR “Eczema” OR “itch”) AND TITLE-ABS-KEY(“sensor” OR “wearable” OR “device” OR “intelligence”)) AND PUBYEAR > 2014
**PUBMED**  (((scratching) AND (itch OR eczema OR pruritus OR “atopic dermatitis”)) AND (sensors OR wearables OR detection OR device OR intelligence))
**Google Scholar**  scratch objective measurement quantify pruritus atopic dermatitis eczema itch sensor wearable device intelligence

**Figure 1:**
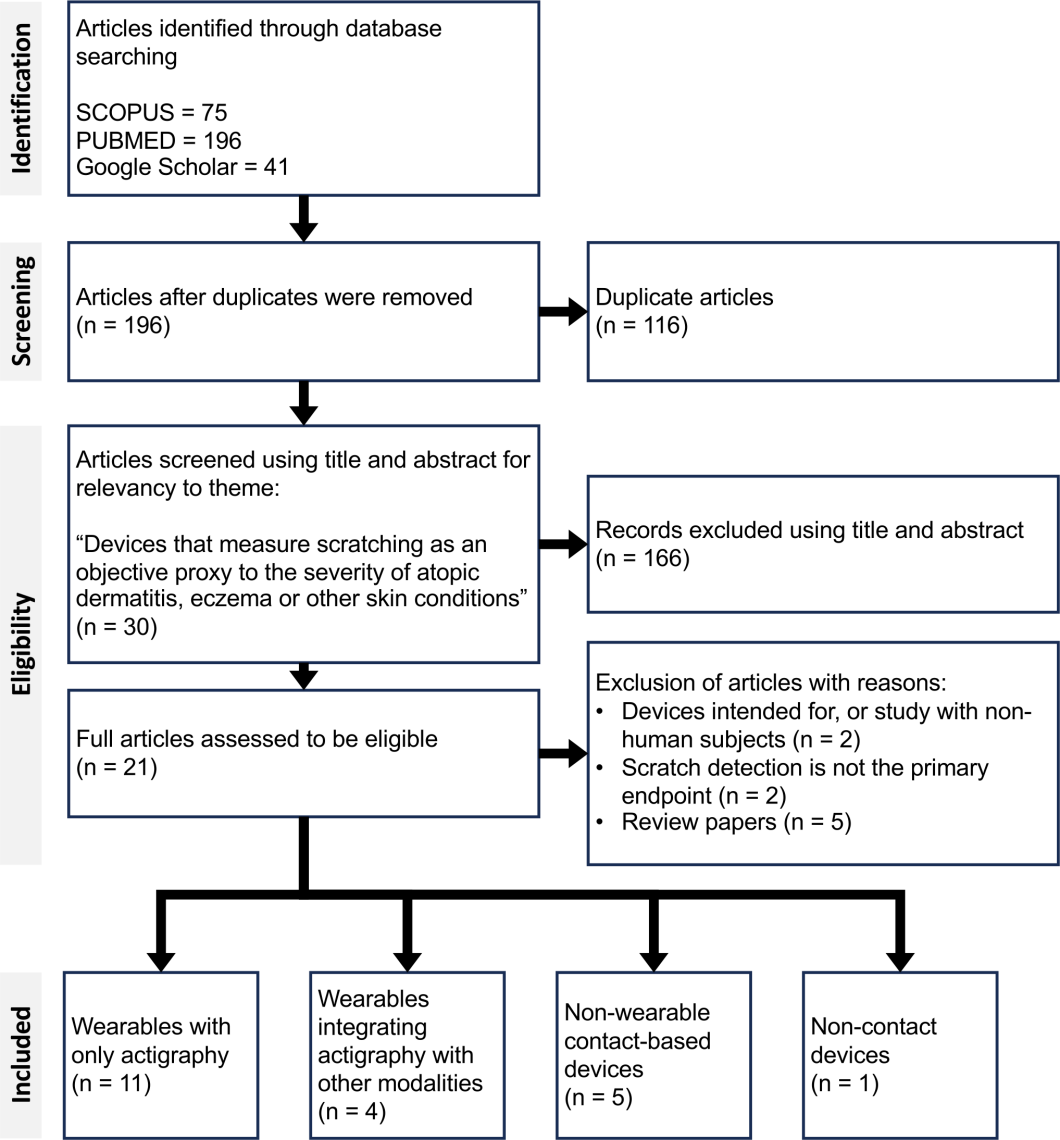
Preferred reporting items for systematic reviews and meta-analyses (PRISMA) flow diagram of the article selection process for this review.

A total of 312 articles were screened for eligibility across three search engines, based on predefined thematic and methodological criteria. Following the screening process, 21 articles were identified that fully met the inclusion criteria (Figure 1). These 21 articles collectively present 14 distinct solutions to scratch detection, each differing in hardware and/or software implementation. These solutions were broadly categorised into four main groups: (1) wearable sensors using only actigraphy, (2) multimodal wearables integrating actigraphy with other type of sensors, (3) non-wearable contact-based devices, and finally (4) non-contact devices. Table 1 summarises studies within Category 1, highlighting the use of IMU-based wearable systems. Table 2 presents the remaining categories (2–4), outlining the technologies employed, their associated performance metrics, and key findings from each article.

## 3. Wearable Sensors for Scratch Detection Using Only Actigraphy

**Figure 2:**
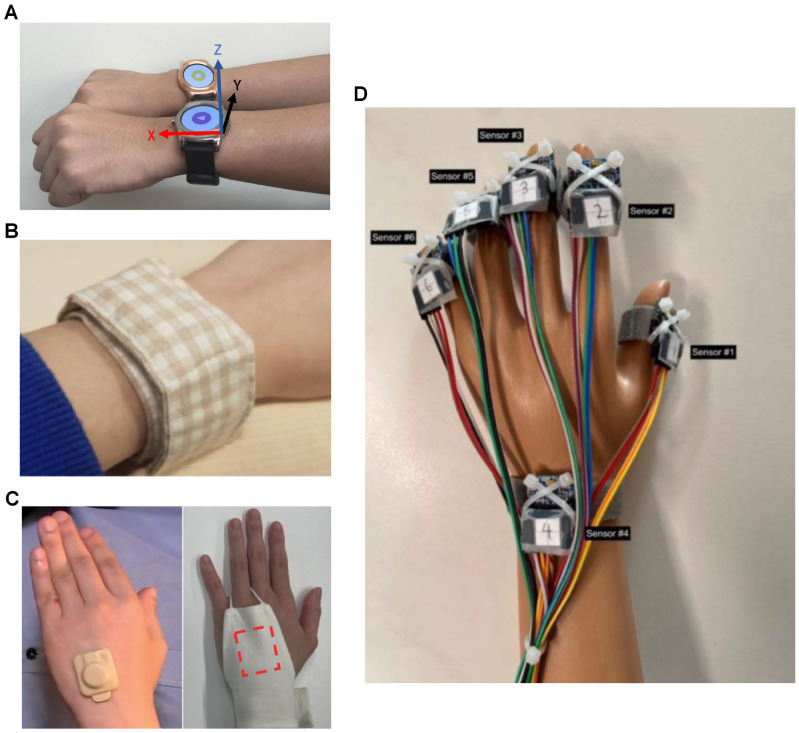
Wearables with only actigraphy, classified into four categories: (**A**) The LG Watch Urbane, a commercial smartwatch integrated with a 3-axis IMU, used for wrist-based motion tracking. Reproduced with permission from the author [21], copyright 2017, ACM, CHI Conference; (**B**) A fabric-based wristband embedded with an IMU device. Reproduced from journal [22], copyright 2021, IEEE SMC; (**C**) Dorsal hand-mounted IMU configuration, where the sensor is attached via an adhesive patch or embedded within a glove. Reproduced from journal [23] under Creative Commons license CC-BY-NC (https://creativecommons.org/licenses/by-nc/4.0/), 2023, Medical Journals Sweden; (**D**) A multi-point IMU system mounted on the fingers. Reproduced with permission from journal [25], copyright 2023, IEEE ICCEICT.

Actigraphy typically utilizes an IMU sensor comprising a gyroscope and an accelerometer embedded within a wearable device (Figure 2). The accelerometer is particularly critical, as scratching is characterized by rapid, repetitive motions of the fingers or forearm, which can be effectively captured by the wearable device when worn on the wrist or hand. Of the 14 solutions identified, 9 employed devices used in actigraphy. Among these, seven utilized commercial off-the-shelf (COTS) hardware paired with custom software implementations for scratch detection, and the remaining two developed bespoke hardware platforms for their implementations.

Ikoma et al. [14] reported the use of the Apple Watch Sport 38 mm (Apple Inc., Cupertino, CA, USA) for scratch detection with their “ItchTracker” application containing a unique algorithm. The first study was to evaluate the performance of the algorithm with five AD patients who participated by wearing a watch on each of their wrists and slept overnight in a video-monitored air-conditioned room. In this case, scratching annotated in the video recordings was graded into “weak”, “moderate”, or “strong” scratching. The detection algorithm works on the fuzzy logic approach by the fulfilment of a series of predetermined thresholds and ranges in the three axes of the accelerometer to differentiate scratching from other movements. Comparing between video annotation and detection with the algorithm, it results in a sensitivity of 0.846, a positive predictive value (PPV) of 0.902, as well as a strong correlation (r = 0.851) for both arms. The second study compared the EASI and ItchTracker-derived scratching duration from a 7-day trial of 30 participants; 20 were AD patients and 10 were healthy, wearing the Apple watch. The result was moderate correlation (r = 0.6) between the clinical assessment score and scratching duration.

The application was further reported in Sugiyama et al. [15] for use with children suffering from AD. Fourteen child participants with one night of wearing the Apple Watch were processed using the ItchTracker algorithm, a strong correlation was attained with EASI for both scratch events per hour (r = 0.7), as well as total scratching time (TST) (r = 0.72). An immediate follow-up study also demonstrated that scratching time became shorter as the EASI clinical assessment score improved.

Xing et al. [16] presented a comparison between three scratch detection models; a convolutional neural network (CNN) model against feature engineering-based approaches using a gradient boosting machine (GBM). The CNN model was made up of three 1-dimensional (1D) convolutional layers, separated by a 1D batch normalisation and a swish activation function. The hidden layer was 128 in size and the adaptive moment estimation (Adam) optimisation technique was used to control the learning rate. The feature engineering approach relied on fast Fourier transform (FFT) to extract the features in the raw accelerometer and gyroscope data as parameters used to train the GBM. Seven participants, five of which were AD patients slept with an Apple Watch (Apple Inc., Cupertino, CA, USA) on each wrist and an infrared (IR) camera for 1 night each for data collection. The post-test validation showed a variety of feature engineering models and performance using data from the device on both hands being the best (Precision = 0.48, Sensitivity = 0.58, Accuracy = 0.97), and it also outperformed the CNN model (Precision = 0.56, Sensitivity = 0.62, Accuracy = 0.93). The explanation for the better performance of the GBM was due to the straightforward conversion of features as input parameters, the ability to leverage engineered features, as well as being less susceptible to overfitting with smaller sample sizes.

Two articles selected COTS medical devices for research, specifically the GeneActiv (Activinsights Ltd., Kimbolton, UK) was chosen by two separate research groups. Moreau et al. [17] utilised the GeneActiv with a long short-term memory recurrent neural network (LSTM-RNN) for their scratch detection algorithm. Their algorithm trained on data from 24 participants, where 18 were AD patients, and spent two to five nights at a sleep laboratory wearing GeneActiv on each of their wrists and were recorded with an IR camera. The LSTM-RNN model performance evaluated with 6-fold cross validation attained a sensitivity up to 0.91 and precision up to 0.89, depending on the participant. The large variation in results was dependent on the participants’ preference of scratching movements, and the two possible contributors were; finger-dominant scratching that the wrist-worn GeneActiv was unable to detect, or scratching movements that produced little motion that the model could not differentiate from background noise. However, when comparing total scratching duration, the model was very strongly correlated (r = 0.945) against video annotation.

Mahadevan et al. [18] presented a study, also using GeneActiv, for monitoring sleep and nocturnal scratching in patients with AD, with the latter of particular interest. This is the same group that published SleepPy, a sleep detection python package using accelerometers [36]. This study involved data from 31 participants for 4 nights each, 2 nights in the sleep clinic where IR video recording was performed and annotated, wearing a device on each of their wrists. The scratch detection module used the random forest (RF) classifier of 50 trees in 3 s windows to balance resolution and performance. The scratch detection module obtained an accuracy of 0.73, sensitivity of 0.61 and specificity of 0.8 when validated with leave-one-subject-out cross-validation (LOSO-CV) after training for both wrists. The module is also very strongly correlated (r = 0.82) for scratch duration, moderately correlated for scratch counts when compared against the video recording. Since this study, the group has published another article using both the sleep and scratch detection modules for a double-blind investigation on the response to abrocitinib treatment, with 11 participants showing the effectiveness of the drug in terms of scratch time and sleep efficiency [37].

Another type of COTS used were multi-sensor logging devices, or data loggers. Ji et al. [19] employed the AX6 data logger (Axivity Ltd., Newcastle upon Tyne, UK) to compare the effectiveness of using accelerometer and gyroscope data, hypothesising that the attitude and angular velocity would be beneficial compared to those that only used accelerometer data. The model used was a LightGBM classifier with input from extracting features, as well as intrinsic topological information. This study collected 96 nights of data from 20 participants with video recording. Comparing the classifiers using LOSO-CV, the addition of the gyroscope data shows improvement to the detection of scratch (Accuracy: Acc = 0.76, Acc + Gyro = 0.78; Sensitivity: Acc = 0.58, Acc + Gyro = 0.64; Specificity: Acc = 0.79, Acc + Gyro = 0.80) due to more precise removal of gravity in the accelerometer data. The classifier-derived scratch time was shorter than the video annotation by an average of 2.13 s. The derived scratch time was moderately correlated to the SCORAD (r = 0.37), as well as weakly correlated to the atopic dermatitis sleep scale (ADSS) (r = 0.22); both fared worse than video annotations (SCORAD: r = 0.42, ADSS = 0.35).

The Itchtector application is a mobile system solution developed for the self-management of itch using actigraphy. Lee et al. [20] first proposed this system using the Samsung Galaxy Gear Live smartwatch (Samsung Electronics, Suwon-si, Republic of Korea) to provide the raw accelerometer data necessary to create the scratch detection software. Three healthy participants spent a night, wearing one watch on each hand, and were recorded with an IR camera for the data. Fifteen different features from the raw data were extracted by cross-referencing with video annotations, and a C4.5 decision tree classifier was used to recognise the scratching. Using 10-fold cross-validation (10F-CV), the mean accuracy of their classifier was 0.969.

Lee et al. [21] is the first article that gave the system their name, it is an extensive and thorough article that laid out the motivations and design principles for Itchtector. The first phase was an investigation of approaches to handle AD and alleviate its symptoms, i.e., itching, by clinicians, as well as by the patient and family. The second phase is the service design of Itchtector; this phase used mock-ups to investigate information required by patients and family to better communicate their condition when they use the Itchtector, as well as parameters that may be relevant to clinicians when understanding their patients’ conditions. They also surveyed patients on the acceptance of the device, wearability, and interest in using it for monitoring their conditions. Based on the user feedback, the group switched to the LG Watch Urbane smart watch (LG Corp., Busan, Republic of Korea) (Figure 2A) for its low weight and long battery life in the third phase. 15 participants were recruited for this phase, and they spent a night wearing the aforementioned smart watch on each of their wrists, as well as being recorded by an IR camera. The classifier used here for scratch detection was an RF classifier, and it attained an accuracy of nearly 0.9, precision of 0.74, and sensitivity of 0.75, also verified with 10F-CV.

A further field trial of the Itchtector application was reported in Kim et al. [22]. The group has swapped out the commercial smart watches for the MetaMotionR data logger (MBientLab Inc., San Jose, CA, USA) (Figure 2B) but with the RF classifier algorithm reported in 2017 remaining unchanged. The main reason for using the MetaMotionR was for the better battery life and weight; the logger was embedded in a fabric wristband. Feedback was collected from the five participants after 14 days of use and the logger was reported to be generally well accepted.

For solutions with custom hardware, Yasuda et al. [23] designed a hand actigraph (HA) (Figure 2C) consisting of only a three-axis accelerometer for attachment to the dorsum of the hand with the use of adhesive tape. This group also worked on a contact-based non-wearable solution, the sheet-shaped body vibrometer (SBV), also reported in this review. The first part of this article reported a study with data from five participants with AD spending a night with the HA on both hands and IR camera. The intention of this study was to validate a modification of a previously reported multi-regression model used for sleep activity [24], now adapted for scratch detection by adjusting the feature extraction methods and equations. Comparing scratch time detected using HA against the video recording, an agreement rate of 0.905, a sensitivity of 0.955 and a specificity of 0.647 were attained and had strong correlation with each other (r = 0.754). The latter part of the article reported an intervention; 40 participants with AD participated for 8 weeks and were provided with one type of moisturiser on top of their treatment regime and the usage was monitored. Nightly results from the HA were also recorded, and the derived parameters were provided to the participants using a smartphone application. The intervention result was a non-statistically significant improvement to their quality of life (QoL) that was assessed with various clinical assessments. Although there were no results from the HA being reported, the authors hypothesised that the participants had more awareness of their conditions and applied the moisturiser properly.

Lastly, Wang et al. [25] introduced a highly customised device featuring six accelerometers positioned on each fingertip, as well as on the wrist (Figure 2D). The author donned two sets of the device on both hands and captured a total of 42 groups of data categorised into eight classes: “face”, “neck”, “left_knee”, “right_knee”, “left_elbow”, “right_elbow”, “others”, and “none”. Using the collected data, seven different deep learning models were designed for scratch detection: “CNN”, “RNN-GRU”, “RNN-LSTM”, “CNN & RNN-GRU (end-to-end)”, “CNN & RNN-LSTM (end-to-end)”, “CNN & RNN-GRU (parallel)”, and “CNN & RNN-LSTM (parallel)”. Each model was customised with computational costs and learning optimisation in mind. Using train–test splitting of 80-20 to validate each model’s performance, the best-performing model was the “CNN“ model with an accuracy of 0.996 in classifying both wrist-dominant and finger-dominant scratching.

Actigraphy is a well researched field with a myriad of references available. There are also a large variety of devices available, from consumer electronics to commercial data loggers, that could be bought and adapted by researchers and research groups that may not have the capability to make their own hardware. All nine solutions presented different types of algorithms but all were able to attain a good level of correlation against video annotations, the gold standard [14,17,18,23], as well as moderate correlation against clinical scores such as SCORAD [19] and EASI [14,15] despite the different combinations of hardware and software solutions. The downsides were false positives [17], and poor sensitivity to finger-dominant scratching [14,17].

## 4. Multimodal Wearable Sensors: Integrating Actigraphy with Additional Scratch-Detection Modalities

**Figure 3:**
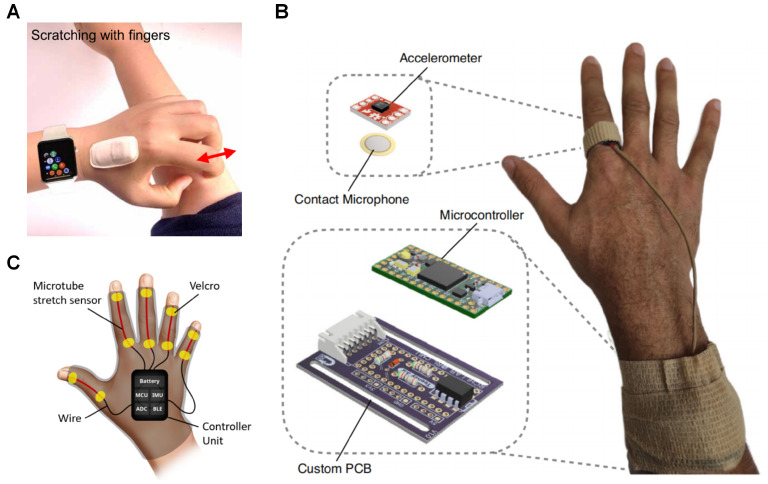
Wearables integrating actigraphy with other modalities: (**A**) An image of a hand-mounted ADAM device and Apple Watch, used to detect scratching movements of wrist and fingers. Reproduced with permission from journal [26], copyright 2021, Science Advances; (**B**) An image of the multimodal sensing ring and its components. Reproduced from journal [28] under Creative Commons license CC-BY (https://creativecommons.org/licenses/by/4.0/), 2023, Communications Medicine; (**C**) A glove-based sensing system (SIGMA) composed of microtube stretch sensors and an onboard IMU. Reproduced from journal [29] under Creative Commons license CC-BY (https://creativecommons.org/licenses/by/4.0/), 2023, MDPI Sensors.

To mitigate the limitations of IMU-based actigraphy, three solutions incorporated additional sensors to work in conjunction with accelerometers and gyroscopes. Among the selected studies, two primary types of supplementary sensors were utilized: strain sensors and acoustic sensors (Figure 3).

The ADvanced Acousto-Mechanic (ADAM) sensor (Figure 3A) is an encapsulated flexible sensor package with an accelerometer and a serpentine acousto-mechanical transducer designed and made in-house, originally conceptualised to detect physiological processes such as seismo-cardiography [38], swallowing, respiration, etc., [39]. Chun et al. [26] reported that the ADAM sensor could also be used to monitor scratching by adhering it to the dorsum of the master hand. An RF classifier of 30 decision trees was trained using various non-scratching data and scratching data from different body locations of 10 healthy participants, and validated using the leave-one-subject-out cross validation (LOSO-CV) (sensitivity = 0.878, specificity = 0.881, accuracy = 0.891). A clinical validation study was also reported with 11 participants of moderate to severe AD in their home environment. The participants were assessed with clinical scores: eczema area and severity index (EASI) and validated investigator global assessments (vIGA), and were provided the ADAM sensor and an IR camera to be used overnight; 46 nights of data were collected and then compared between ADAM and the IR camera (sensitivity = 0.843, specificity = 0.993, accuracy = 0.99).

The high specificity attained was due to scratch-generated sounds being detected with the acousto-mechanical transducer. A report with a new AI-algorithm that could quantify scratching with key sleep metrics was presented in Yang et al. [27], it was validated with 73 nights of data yielding a sensitivity of 0.93 and a specificity of 1 against an IR camera. When assessing against sleep metrics, it was found to have moderate correlation with scratch events (SE) per hour, as well as scratch events per night against total sleep time (SE/h: r = 0.69, SE/night: r = 0.54).

Padmanabha et al. [28] demonstrated a device similar to ADAM with an accelerometer and a vibration contact microphone in a ring worn on the index finger of the dominant hand of the user (Figure 3B). In addition to being able to detect scratching, it was also able to estimate the scratch intensity from scratch-generated sounds. In the study, 20 healthy participants scratching at various intensities were used to train two neural networks with ReLU activation: one for detecting scratching and the other for intensity with different layer size and amount hidden layers. The two hidden layers with a layer size of 1000 nodes for scratch detection (Accuracy = 0.8998), and the three hidden layers with a layer size of 1200 nodes were validated using LOSO-CV to have an averaged mean absolute percentage error (MAPE) of 0.0829 using both accelerometer and contact microphone as input. The intensity estimation was also validated against a pressure-sensitive tablet (Morph, Sensel Inc, Sunnyvale, CA, USA) in a separate study with 14 healthy participants and found to have MAPE of 0.0957 and strong correlation (r = 0.83).

Au et al. [29] introduced SIGMA, a wearable glove equipped with flexible strain sensors embedded in each finger and an IMU placed on the dorsum of the hand (Figure 3C) with a custom LSTM-CNN model trained using a dataset of scratching and rubbing of various intensities, as well as a variety of non-scratching actions from five participants and tested using train–test splitting (Sensitivity (Sens) = 0.83, specificity (Spec) = 0.84, and accuracy (Acc) = 0.83). The validation was done in the form of a 30-min study with a fixed set of actions that were different from the training set at known intervals. In this scenario, SIGMA demonstrated high specificity (0.99) and accuracy (0.99), but its sensitivity was lower (0.75), with a particular improvement reported for detection of finger-dominant scratching. SIGMA was reported to not be able to reduce false positives, particularly with actions that mimicked scratching such as “open and close hands in air“, one of the actions in the validation study. Additionally, an unconstrained 30-min pilot study during awake hours with eight child participants diagnosed with atopic dermatitis (AD) was reported. In the pilot study, SIGMA showed a significant drop in sensitivity (0.40) due to a large number of false positives from voluntary hand movements. The awake hours study highlighted the limitations of using scratching as an objective measure when users have voluntary control over their hand movements, leading to substantial reductions in sensitivity. This article is the first article found with a scratch study where the patients were awake throughout.

All three multimodal wearables reviewed also relied on machine learning models and were trained on a diverse set of scratching actions of varying intensities, as well as non-scratching actions, and were presented as able to detect scratching with high sensitivity, specificity, and accuracy. Both ADAM and the multimodal sensing ring used contact-based transducers which would be incapable of capturing speech or audio. The performance when users were awake was also demonstrated to be suboptimal [29], but demonstrated excellent performance, especially in terms of specificity, in a nocturnal setting when validated against video recordings [26,27], as well as strong correlation [27]. Additionally, Padmanabha et al., 2023 reported that their multimodal sensing ring was also capable of estimating scratching intensity with very low percentage error and showed a strong correlation with measurements obtained from a commercial pressure-sensing tablet [28] while having similar accuracy to ADAM (Sensing ring: 0.8998, ADAM: 0.93).

## 5. Non-Wearable Contact-Based Scratch Detection

**Figure 4:**
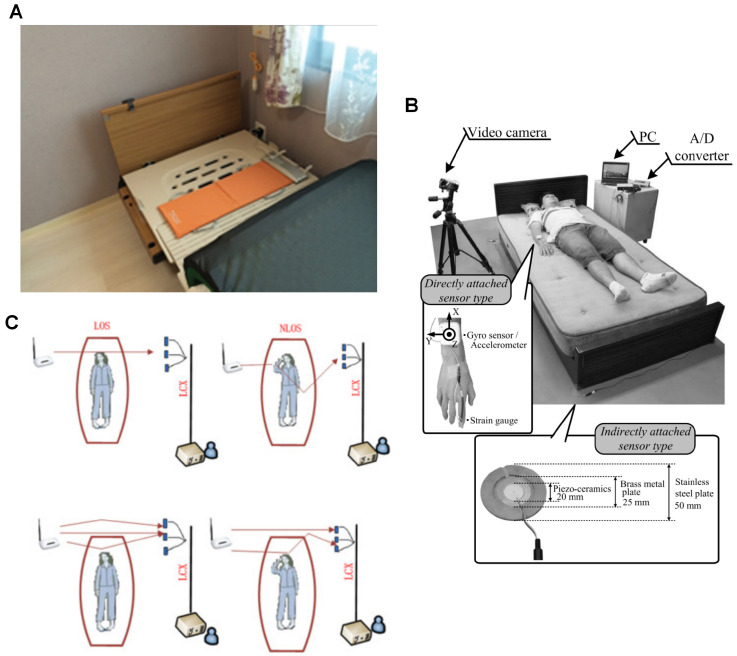
Non-wearable contact-based (**A**,**B**) or non-contact systems (**C**) for monitoring scratching behaviour and sleep-related activity: (**A**) An SBV integrated into the bed surface. Reproduced with permission from the author [30], copyright 2018, Journal of Clinical Sleep Medicine; (**B**) The vibration sensing system setup with video camera and strain gauge actigraph (top), the dimensions of a piezoceramic sensor of the system (bottom). Reproduced with permission from journal [34], copyright 2017, IEEE Sensors Journal; (**C**) An illustration of the LCX setup changes in communication paths when scratching. Reproduced with permission from journal [35], copyright 2017, Healthcare Technology Letters.

Two solutions were identified that do not require users to wear any devices. Instead, these systems detect movements that reverberate across the user and objects in contact with them. Over the past decade, these solutions have undergone significant advancement, driven by multiple publications. Originally developed for other applications, the research groups behind these solutions began exploring their potential for detecting nocturnal scratching (Figure 4). As a result, specialised algorithms were developed to adapt these systems specifically for scratch detection.

The sheet-shaped vibrometer (SBV) (Figure 4A), marketed as NEMURI SCAN (Paramount Bed Ltd., Tokyo, Japan), is an array of pressure sensors placed under the mattress to detect motion and vibrations for sleep activity monitoring [24]. Originally developed for sleep activity detection, the SBV has also been utilized for screening obstructive sleep apnea [40] and for neonatal care in intensive care units [41].

Kogure et al. [30] first adapted the SBV for nocturnal scratching detection, employing the same algorithm initially developed for sleep activity monitoring. This algorithm used a multiple-regression model based on the Cole–Kripke equation [24]. In their study, the SBV was compared to a wearable actigraph (WA) (Philips Respironics, Murrysville, PA, USA) using paired overnight measurements from 20 participants with AD. The results revealed that the SBV performed worse than the WA when correlating against the clinical SCORAD score (SBV: r = 0.431, WA: r = 0.641), despite a moderate correlation between the two devices (r = 0.633). The article concluded that the SBV was not designed to differentiate scratching and required further refinement for this purpose.

This issue was addressed by Toyota et al. [31], who developed a dedicated scratch detection algorithm for the SBV. The new algorithm enhanced specificity by analysing activity counts based on frequency and amplitude, rejecting non-scratching body movements and heart rate interference [41]. In a follow-up study with 10 participants (7 with AD), the new algorithm was evaluated alongside the original sleep activity algorithm, using an IR camera for comparison and 73 paired overnight measurements. The results showed the newly developed, dedicated algorithm had a significantly better correlation with the IR camera (New: r = 0.89, Old: r = 0.72). Additionally, both algorithms were compared against two clinical scores, EASI and NRS. The new algorithm demonstrated improved correlation with both scores (EASI: New: r = 0.5, Old: r = 0, Camera: r = 0.5; NRS: New: r = 0.42, Old: r = 0.18, Camera: r = 0.42), with correlations comparable to those of the IR camera.

The vibration sensing system (VBS) initially introduced in Nukaya et al., 2012 [42,43] consisted of four piezoceramic sensor modules placed between the bed legs and the floor (Figure 4B), originally designed to monitor human biosignals such as respiration and heart rate. This system was subsequently adapted to detect high-cyclical scratching motions, with a newly developed mathematical model that provided visually perceivable outputs for scratching [44]. In a study with a predefined scratching protocol (35 sets of 20 cheek scratches, 5-s intervals), the root-mean-squared error (RMSE) of TST, when compared to camera recordings, was found to depend on the distance between the sensor and the scratching point. The error was minimized when the sensor was placed closest to the scratching location (RMSE = 0.87–6.31% TST) [45].

Kurihara et al. [32] further enhanced the system by incorporating wide dynamic range piezoceramic sensors with the goal of identifying multiple actions, including “scratching,” “turning over,” and “sitting up/lying down.” As the latter two actions produced significantly larger vibrations than scratching, the team validated the sensitivity of the new sensors. The model was also upgraded to distinguish between different turning directions and sitting/lying postures. In their validation study, a participant performed the same scratching actions as in the previous study while wearing a strain gauge on the index finger of the dominant hand. Results showed visual concordance between the vibration-sensing system and the strain gauge, confirming the system’s ability to accurately detect scratching movements.

A clinical validation with 12 healthy participants was conducted in Kurihara et al. [33], where three different scratching types were evaluated: 35 sets of 20 cheek scratches, 20 back scratches, and 10 shin scratches, all performed using the right foot. The RMSE for each action was again found to be dependent on the sensor-scratching distance: cheek scratches (RMSE = 0.56 s), back scratches (RMSE = 0.83 s), and shin scratches (RMSE = 1.29 s). The system was also able to identify other actions, such as turning over and abnormal hand/foot movements. The overnight evaluation confirmed the system’s stability and presented detailed profiles of the three scratching actions evaluated during the test.

To further enhance the system’s performance, Kaburagi et al. [34] introduced an algorithm to automate the detection of TST. The algorithm utilized a moving average filter to eliminate natural bed vibrations, cut off low-frequency motions (such as arm and leg movements), analysing the remaining signal amplitude to isolate scratching from other noise sources like heart rate. Additionally, a version of the algorithm was proposed for use with a strain gauge on the index finger and an IMU on the dorsum of the hand, focusing on amplitude analysis only. A validation study with 12 healthy participants using the same cheek scratch protocol as in previous studies showed that the new algorithm reduced RMSE %TST compared to manual identification (Manual: 1.84–6.83% TST, Algorithm: 0.92–6.58% TST). The best performance was again observed when the sensor was placed on the right side of the head of the bed where the distance to the scratch site is closest. The algorithm also improved the detection accuracy by reducing misjudgment of body movements as scratching, a common issue with manual identification.

The non-intrusive nature of the contact-based sensing solutions ensured that they remained securely in place during use, preventing issues such as device dislodgement [23,32] or potentially aggravating the user’s condition. Additionally, these solutions addressed the common issue of forgetfulness, as users would not need to remember to wear a device [30]. However, these solutions come with certain limitations; the systems require setup, entailing users to be on the prepared bed for detection to take place. Furthermore, the systems are unable to distinguish between multiple users, limiting their use to a single individual at a time. Additionally, the performance of the sensor is highly dependent on the proximity of the scratching motion to the sensor [45], which could result in additional costs to provide coverage across a bed. When functioning optimally, the systems exhibit high specificity in detecting scratching motions, especially when enhanced by specially designed algorithms that reduce overestimation of scratching behaviour [31,34]. However, concerns remain regarding non-scratching actions that produce similar waveforms, potentially leading to false positives [31]. Despite these concerns, both solutions presented have had demonstrated excellent performance, with low error rates in TST [33,34] and strong correlations with video camera recordings [30,31]. They also show moderate correlations with clinical scores such as SCORAD [30] and EASI [31]. A notable advantage of these solutions are that they do not require machine learning algorithms to achieve these results and do not suffer from blind spots where scratching motions could go undetected.

## 6. Non-Contact Solutions for Scratch Detection

Non-contact sensing technologies, unlike the previously discussed solutions, do not require physical contact between the user and the sensor. These technologies typically rely on electromagnetic, sonic, or optical signals, which are interpreted remotely by a device. One of the most established and accepted gold standards for objectively detecting scratching is the use of IR cameras [11], a non-contact solution. In the past decade, only one article was identified that explored a non-visual, non-contact solution for scratch detection.

Dong et al. [35] introduced a novel non-contact scratch detection technology using a router-based leaky coaxial cable (LCX) slotted receiver system (Figure 4C). This system detects movements by analysing the attenuation of the IEEE 802.11n [46] channel state information (CSI) package transmitted by a 2.4 GHz Wi-Fi router. The article demonstrated the system’s capabilities using both line-of-sight (LOS) and non-line-of-sight (NLOS) propagation methods, highlighting the system’s sensitivity to detecting movements, including scratching, through a multipath approach. In a small-scale experiment with only two healthy participants, a total of 35 sets of data were collected, each set consisting of predefined actions, including 20 cheek scratches with a 5 s interval. The results showed that movements caused a shift in phase in the received CSI package. More notably, analysis of the CSI subcarrier trend revealed visually distinct profiles between arm-to-cheek movements and scratching motions. This finding suggests that the system exhibits high specificity in distinguishing scratching from other arm movements.

With non-contact solutions, the main advantage is that users do not need to be physically in touch with the detection modality. The LCX also possesses fewer blind spots and better reliability than the gold standard due to multiple propagation signal paths that detect the movements within the detection field. Conversely, the multiple propagation paths can also negatively distort received signals, especially in rooms with items that have high reflectance causing “echo” signals to be received by the receiver, complicating the signal processing. The LCX setup is also more sophisticated than the VBS or SBV in that both hardware and software require a wholly different, specialised domain knowledge to be able to design it, which may explain the sole article in the last decade and the lack of clinical validation. Similar to contact-based non-wearable devices, there is an element of setting up a detection field that only works with a single person; the wireless detection solution presented was incapable of separating between multiple people within the field of view.

## 7. Conclusions

The availability of high-quality, reliable commercial off-the-shelf (COTS) wearable devices has significantly lowered the barrier for clinicians and research groups. By leveraging COTS devices, research groups can focus more on developing specialised machine learning algorithms without the need to design custom hardware from scratch. This has contributed to an increase in publications over the past decade, in particular in actigraphy-based wearables. However developing bespoke multimodal wearable systems, or the more complex contact-based and contactless non-wearable solutions requires expertise in multiple domains that few research groups possess; expertise involved in hardware design, algorithm development and training, as well as access to patient populations for validation. Even among capable groups, such as those who developed the VBS system, advancing the bespoke solutions to the point of clinical validation is time- and resource-intensive. That said, building a custom device offers unique advantages as it enables the integration of multiple sensing modalities or a different setup, which leads to potential improvements in selectivity, user comfort, and adherence.

Another key insight from this review is the lack of standardisation in algorithm selection and validation methodology. This issue has led to a wide range of model algorithms used with varying performance. To address this, there is a need for a modular, preferably open-source scratch detection framework that has a multitude of machine learning models that is also capable of real-time processing. Such a framework would be analogous to the accessibility provided by COTS hardware, to assist research groups with hardware development capabilities to build effective scratch detection models for atopic dermatitis(AD) management.

This review has explored and reported on the development of a range of devices that use scratching as an objective proxy of AD. The solutions presented serve as examples for researchers and developers to create a scratch detecting solution aimed at addressing the privacy issues and labour-intensive processing of video recordings using other sensor modalities, with the assistance of machine learning algorithms. While the focus is to reduce workload in clinical settings, successful automation of scratch detection would also pave the way for consumer-grade solutions for AD management.

## Figures and Tables

**Table 1:** Summary table for articles that report on scratch detection using only actigraphy.

Reference	Technology	Participants	Sensitivity	Specificity	Accuracy	Correlation
Ikoma [14]	Apple Watch with Itchtracker (fuzzy logic)	5 AD (Train); 20 AD, 10 Healthy (Study)	0.846 (vid)			r = 0.851 [both arms] (vid), r = 0.6 (EASI)
Sugiyama [15]		14 AD (Study)				r = 0.70 (SE/hr vs. EASI), r = 0.72 (TST vs. EASI)
Xing [16]	Apple Watch with CNN and Classifier (GBM)	5 AD, 2 Healthy	0.58 [GBM], 0.62 [CNN] (post-train); 0.46 [Classifier, both hands], 0.63 [CNN] (vid)		0.97 [GBM], 0.93 [CNN] (post-train); 0.84 [Classifier, both hands], 0.72 [CNN] (vid)	
Moreau [17]	GeneActiv with LSTM-RNN	18 AD, 6 Healthy	0.0-0.91(6F-CV)			r = 0.945 (vid)
Mahadevan [18]	GeneActiv with Classifier (Random Forest)	31 AD	0.61 (LOSO-CV)	0.8 (LOSO-CV)	0.73 (LOSO-CV)	r = 0.82 (vid)
Ji [19]	AX6 with Classifier (LightGBM)	20 AD	0.58 [accel]; 0.64 [accel + gyro] (LOSO-CV)	0.79 [accel]; 0.8 [accel + gyro] (LOSO-CV)	0.76 [accel]; 0.78 [accel+gyro] (LOSO-CV)	r = 0.37 [derived] (SCORAD), r = 0.22 [derived] (ADSS)
Lee [20]	Itchtector app on Android smartwatches with Classifier (C4.5 decision tree)	3 Healthy			0.969 [Mean] (10F-CV)	
Lee [21]	Itchtector app on Android smartwatches with Classifier (Random Forest)	11 AD, 4 Others	0.75 (10F-CV)		0.9 (10F-CV)	
Kim [22]	Metamotion with Classifier (Random Forest)					
Yasuda [23]	Hand Actigraph with Multi-regression [24]	5 AD	0.995 (vid)	0.647 (vid)		r = 0.754 [both hands] (vid)
Wang [25]	New accelerometer sensor layout design with 7 deep learning models	1 Healthy			0.996 [CNN] (post-train)	

Note. AD = Participants affected with skin disease, ADSS = Atopic dermatitis sleep scale, EASI = Eczema Area and Severity Index, GBM = gradient boosting machine, LOSO-CV = leave-one-subject-out cross-validation, Others = Participants with various itching conditions, SCORAD = Severity Scoring of Atopic Dermatitis Index, SE = scratch event, TST = total scratch time, 6F-CV = 6-fold cross-validation, 10F-CV = 10-fold cross-validation.

**Table 2:** Summary table for articles that report on scratch detection using wearables integrating actigraphy with other modalities, non-wearable contact-based devices, as well as non-contact devices.

Reference	Technology	Participants	Sensitivity	Specificity	Accuracy	Correlation
Chun [26]	ADAM with Classifier (Random Forest)	10 Healthy (Train), 11 AD (Study)	0.878 (LOSO-CV); 0.843 (vid)	0.881 (LOSO-CV); 0.993 (vid)	0.891 (LOSO-CV); 0.99 (vid)	
Yang [27]	ADAM with Classifier (Random Forest) [26]	10 AD (Study)	Sens = 0.93 (vid)	Spec = 1 (vid)		r = 0.69 (SE/h vs. TST), r = 0.54 (SE/night vs. TST)
Padmanadha [28]	Multimodal sensing Ring with ReLU-NN	Intensity: 20 Healthy (Train), 14 Healthy (Study); Detection: 20 Healthy (Train)			Detection: 0.8998 (LOSO-CV)	Intensity: MAPE = 0.0829 (LOSO-CV), MAPE = 0.0957 (tablet); r = 0.83 (tablet)
Au [29]	SIGMA with LSTM-CNN	5 Healthy (Train, 30 min), 8 AD (Study)	0.83 (train-test split); 0.74 (30 min); 0.4 (vid)	0.84 (train-test split); 0.99 (30 min); 0.84 (vid)	0.83 (train-test split); 0.99 (30 min); 0.83 (vid)	
Kogure [30]	SBV with Signal Processing	20 AD (Study)				r = 0.633 (actigraphy); r = 0.431 (SCORAD)
Toyota [31]	SBV with Signal Processing	7 AD, 3 Healthy (Study)				r = 0.72 [Old], r = 0.89 [New] (vid); r = 0.5 [EASI], r = 0.42 [NRS]
Kurihara [32]	Vibration sensing system with Signal Processing					
Kurihara [33]		1 Healthy				RMSE = 0.56–1.29 sec (vid)
Kaburagi [34]		1 Healthy				RMSE = 1.84–6.83 %TST [Manual]; RMSE = 0.92–6.58 %TST [Algorithmic] (vid)
Dong [35]	Leaky Coaxial Cable (LCX) with Signal Processing					

Note. AD = Participants affected with skin disease, ADAM = ADvanced Acousto-Mechanic, EASI = Eczema Area and Severity Index, LOSO-CV = leave-one-subject-out cross-validation, MAPE = mean absolute percentage error, NRS = Numeric Rating Scale, RMSE = root-mean-squared error, SBV = Sheet-shaped body vibrometer, SCORAD = Scoring Atopic Dermatitis, SE = scratch events, TST = total sleep time.
